# Prevalence of early skin-to-skin contact and its impact on exclusive breastfeeding during the maternity hospitalization

**DOI:** 10.1186/s12887-022-03455-3

**Published:** 2022-07-07

**Authors:** Hoang Thi Nam Giang, Do Thi Thuy Duy, Nguyen Lam Vuong, Nguyen Thi Tu Ngoc, Thu Thi Pham, Le Quang Tuan, Le Oai, Pham Do Thuc Anh, Ton That Khanh, Nguyen Thi Anh Thi, Mai Ngoc Luu, Tran Thi Tuyet Nga, Le Tho Minh Hieu, Nguyen Tien Huy

**Affiliations:** 1 School of Medicine and Pharmacy, The University of Danang, Danang City, Vietnam; 2 University of Medicine and Pharmacy at Ho Chi Minh City, Ho Chi Minh City, Vietnam; 3 Thai Nguyen University of Medicine and Pharmacy, Thai Nguyen City, Vietnam; 4 Department of Internal Medicine, University of Medicine and Pharmacy at Ho Chi Minh City, Ho Chi Minh City, Vietnam; 5 School of Tropical Medicine and Global Health, Nagasaki University, Nagasaki, Japan

**Keywords:** Skin to skin contact, Breastfeeding, Early Essential Newborn Care, Risk factors, Dose–response relationship, Hospital

## Abstract

**Background:**

Early essential newborn care has been implemented in countries regardless high or low neonatal mortality. This study aims to investigate the current practice of skin-to-skin contact (SSC) and its effect on exclusive breastfeeding during the hospital stay.

**Methods:**

This is a cross-sectional study of 1812 Vietnamese mothers in multicenter. A questionnaire answered by the mothers was used to assess the duration of both SSC and breastfeeding practices. Multivariable logistic regression was used to identify a dose–response relationship between early SSC and prevalence of exclusive breastfeeding in hospital.

**Results:**

There were 88.7% of mothers experiencing SSC with their infants right after birth and the highest prevalence of SSC was found in district hospitals. Among those experiencing SSC, 18.8% of the infants received more than 90 min of SSC and completed the first breastfeeding during SSC time. Prevalence of exclusive breastfeeding during maternity hospital stay was 46.7%. We found a significant dose–response relation between the duration of SSC and exclusive breastfeeding in hospital. Compared with infants without SSC, the prevalence of exclusive breastfeeding was higher in infants who experienced SSC for 15–90 min (adjusted odds ratio [aOR], 95% confidence interval [95%-CI]: 2.62 [1.61–4.27]) and more than 90 min (aOR [95%-CI]: 5.98 [3.48–10.28]). Completed first breastfeeding during SSC time (aOR [95%-CI]: 4.24 [3.28–5.47]) and being born in district hospitals (aOR [95%-CI]: 2.35 [1.79–3.09]) were associated with increased prevalence of exclusive breastfeeding during hospital stay. On the other hand, mother education level as high school/intermediate (aOR [95%-CI]: 0.58 [0.42–0.82]) and place of residence classified as rural decreased odds of exclusive breastfeeding in hospital (aOR [95%-CI]: 0.78 [0.61–0.99]).

**Conclusion:**

Our results demonstrate a strong dose–response relationship between duration of SSC and exclusive breastfeeding in hospital. Interventions that support exclusive breastfeeding during hospital stay, especially achieving prolonged uninterrupted SSC, could improve the duration of breastfeeding.

## Introduction

Optimal breastfeeding practice is one of the most cost-effective intervention for improving mortality and morbidity for children under five years [1, 2]. It could save over 800,000 or 12% of all deaths annually [3, 4]. Despite strong evidence of the short- and long-term health benefits for both children and mothers, at global level, nearly two third of infants are not exclusively breastfed during the first six months of life and nearly 60% of infants are not put on breast in the first hour of life [5]. Strategies that have had great impact on promoting breastfeeding rate, especially in developing countries, includes breastfeeding education and support. Skin-to-skin contact (SSC) is another crucial evidence-based practice that provides a maximized chance for breastfeeding [6, 7].

Early exclusive breastfeeding during hospital stay was identified as an effective strategy to improve the duration of breastfeeding [8–10]. Infants who breastfed exclusively in hospital were breastfed for four months longer than those who supplemented with formula [8]. Among mothers who intended exclusive breastfeeding, by day 60, formula supplementation in hospital was associated threefold risk of cessation of breastfeeding [10]. Thus, improving exclusive breastfeeding during hospital stay could have significant impact on short- and long-term breastfeeding outcomes. Despite the benefits, the proportion of exclusive breastfeeding in hospital remains lower than the current recommendations. Vehling et al. showed a prevalence of 74% infants were exclusively breastfed in hospital in Canada [8], Cox et al. reported a prevalence of 82.7% in Australia [11], while this proportions were lower in areas of southern Vietnam with 33% [12]. Identifying factors associated with exclusive breastfeeding in hospital is important to prioritize strategies for promoting exclusive breastfeeding in hospital.

Immediate SSC is referred to the placement of the naked baby prone to the bare chest of his or her mother at birth. Prolonged SSC is an important component of the package intrapartum and immediate newborn care interventions named Early Essential Newborn Care (EENC). This package includes: immediate and thorough drying newborn; immediate and sustained SSC between mother and babies for at least 90 min; delayed cord clamping for at least one to three minutes; non-separation mother and babies until the first breastfeeding is completed [13]. Since 2014, World Health Organization (WHO) has supported countries in the Western Pacific Region to scale up EENC widely. During the COVID-19 pandemic, SSC is still recommended by WHO as “the benefits substantially outweigh the potential risks of transmission and illness associated with the disease” [14]. Despite strong recommendations from the WHO, the practice of SSC varies from 1–98% of infants across the world, with higher prevalence of SSC in high income countries compared to low-middle-income countries with a range from 8 to 74% [15]. Variation in the starting time and duration of SSC is another concern. Nevertheless, limited number of studies have been reported in developing countries to identifying the rate of SSC and its effects [15]. Several studies with small sample size showed evidence support the benefits of SSC to breastfeeding at hospital discharge and one to four months after birth [16]. Women who experienced SSC also breastfed their children longer [16]. Further, Bramson 2010 and Li 2020 found that a longer duration of SSC was correlated with a higher rate of exclusive breastfeeding [17, 18]. However, there are still lacking evidence on clear advantages of longer duration of SSC and optimal duration of SSC has not been established yet.

Between 2016 and 2017, among 1383 maternal interviews from eight countries in East Asia and the Pacific, 90.9% of mothers practiced SSC with their babies after birth [18]. The exclusive breastfeeding rate since birth and before discharge ranged from 67.8% for infants not experienced SSC to 92.7% for infants placed in 60–90 min of uninterrupted SSC. On the other hand, exclusive breastfeeding rate at 4–5 months was 41.3% for this region between 2010 and 2018 [19]. Vietnam is a country in the Western Pacific Region that adopted EENC in 2014. In 2017, about 88% of hospitals in all three levels of public hospitals (central, provincial, and district levels) started to implement EENC [20]. Currently, there is no study showing the prevalence of SSC in Vietnam. Report from the Vietnam Ministry of Health and WHO in 2017 showed that 59% of all newborns received SSC immediately after birth and 57% of infants were breastfed before separation from mothers [18, 20]. The major limitation of these reports is the small sample size [18]. Also, different method of data collection results in different breastfeeding rates. Binns et al. showed a large discrepancy between breastfeeding rate in Western Pacific Region reported by national representative data of WHO and UNICEF and the rate reported by a cohort study [21]. Our current study with a large sample size focusing not only on prevalence but duration of SSC and its impact on breastfeeding outcomes in a lower middle-income country. This study aims to investigate the current practice of SSC and its effect on exclusive breastfeeding during the hospital stay.

## Methods

### Study setting

This study was conducted in Da Nang City and Thai Nguyen City, Vietnam. Da Nang is the largest city located in the central of Vietnam with a population of over 1.1 million in 2020, in which nearly 90% of the population residing are in urban areas. The city is officially divided into 45 wards and 11 communes [22]. In 2014, Da Nang Hospital for Women and Children was among the first three teaching hospitals in Vietnam to introduce EENC [13]. At present, all hospitals in Da Nang City implemented EENC. Thai Nguyen City is a largest city of Thai Nguyen province, located in the Northeast region of Vietnam. The city is officially divided into 21 wards and 11 communes. EENC was introduced in Thai Nguyen in 2015. In Vietnam, the average length of hospital stay after a normal vaginal delivery and caesarean section (C-section) are three to five days.

In Vietnam, health care system consists of public and private sector. Public health care system are divided into four administrative levels: central level, provincial level, district level and commune level [23]. Over-crowding is a major problem in central and provincial levels [23]. Commune Health Center (CHC) provides a wide range of primary health care services such as maternal, child health care and immunization.

### Study design and eligible participants

A cross sectional study using a self-administered questionnaire was conducted from March to May 2021 to assess the practice of breastfeeding from birth to 24 months of age. Mothers were invited to join the study when they brought their children to the CHC for routine immunisation. If the children were under 30 months of age, the mothers of singletons were eligible for the study. Current report of breastfeeding practices during the maternity hospitalization used data from this study.

### Study procedure and sampling process

**Fig. 1:**
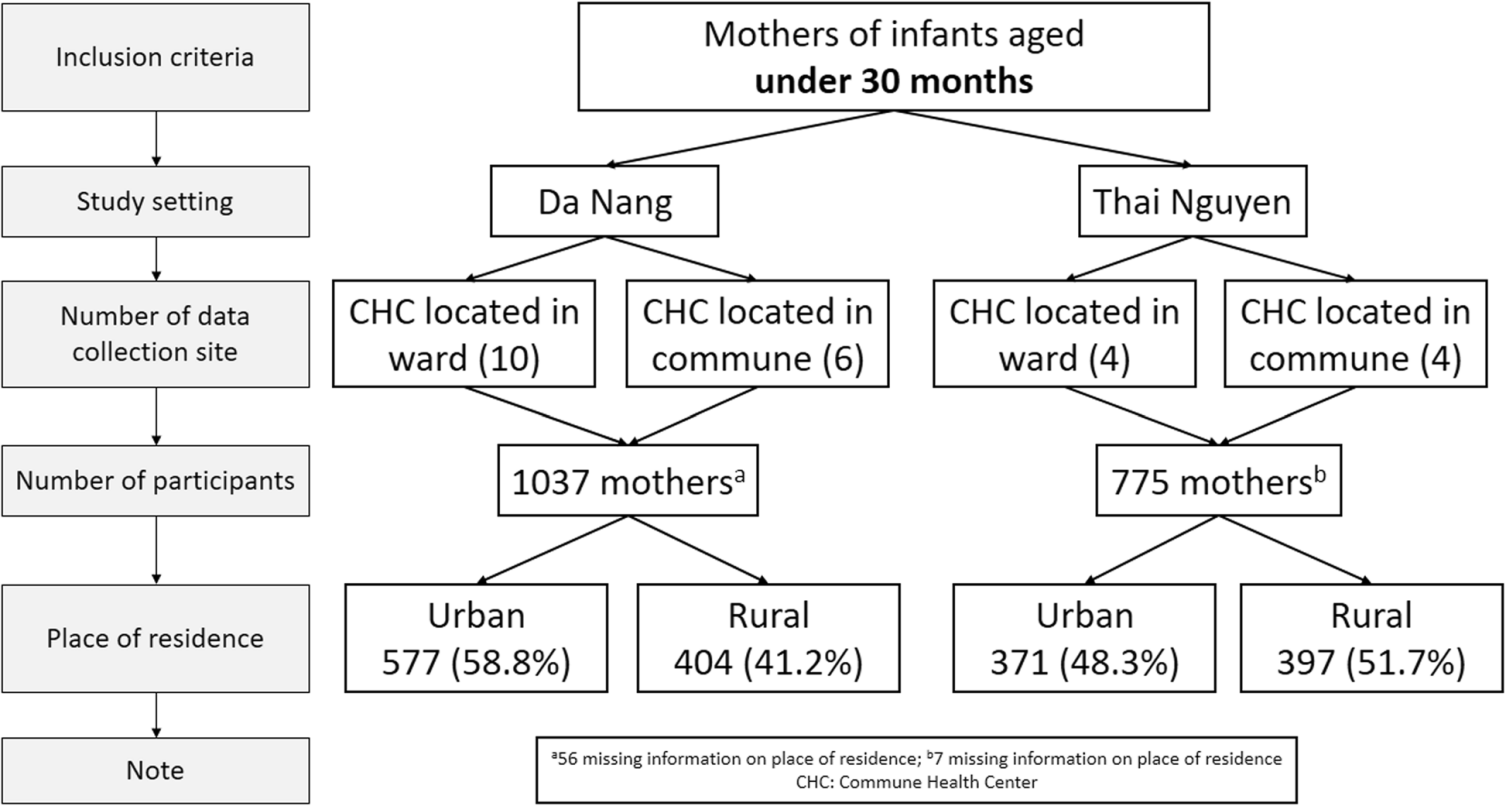
Sampling procedure and study participants

One key study member in each city managed the data collection. These researchers were responsible for training data collection for data collectors in Thai Nguyen and Da Nang. In Thai Nguyen, recruitment was conducted at eight CHCs, four located in urban areas and four in rural areas. In Da Nang, recruitment was conducted in 10 CHCs located in the urban areas and six located in the rural areas. On the day of immunization of National Expanded Programme on Immunization, all parents are recommended bringing their children to the CHC. The data collectors approached and invited all mothers who had infants aged under 30 months to join the study. The detailed sampling procedure is described in Fig. 1.

The first question in the questionnaire was designed as a written informed consent after mothers provided verbal informed consent. The data collectors distributed the printed questionnaires to mothers and collected the completed questionnaire when mothers finished. Data collection was approved by leaders of CHCs in advance.

### Sample size calculation

The sample size was calculated by using formula of estimating prevalence in cross sectional studies considering the following assumptions: Prevalence of exclusive breastfeeding during hospital stay 33.0% [12], 95% of confidence level, precision error of 2.2% and 5% non-response rate. The required sample size should be 1843 mothers.

### Questionnaire

The questionnaire was developed after reviewing literature and consulting with experts on breastfeeding and EENC practices. The original questionnaire included 39 questions. Current report used information on 13 questions which were grouped in four categories: (i) maternal characteristics included age, highest education level (primary school, secondary school, high school/ intermediate degree, university/ postgraduate), place of residence, complication during pregnancy and at birth, parity, delivery method (vaginal or C-section), type of hospital of birth, (ii) infant characteristics covered sex, birth weight, current age of the infants and complication at birth, (iii) breastfeeding practices questioned mothers in feeding their children during maternity hospital stay which included breast milk (yes/no), formula (yes/no), colostrum milk powder (yes/no), water (yes/no), fruit juices (yes/no), (iv) SSC practices (yes/no) and the amount of time spent on SSC (< 15 min, 15–90 min, > 90 min) and receiving first breastfeeding during the time of SSC (yes/no). We asked mothers if the baby was placed directly on their chest—“skin to skin contact” right after birth. For those who answered “yes”, we asked them to estimate the amount of time spent on SSC by choosing one of three categories < 15 min, or 15–90 min, or > 90 min. According to previous study, the proportion of Vietnamese mothers discarded their colostrum was high at 37% as it is thought that colostrum is not good for babies [24]. Therefore, in many cases, in addition to breast milk or formula, babies are supplemented with colostrum milk powder in the first few days of life. In order to describe the using of colostrum milk powder in detail, the analysis was performed separately for formula and colostrum milk powder. This does not affect the reported exclusive breastfeeding rate.

Pilot testing of the original questionnaire was conducted in 10 mothers who met the inclusion criteria. The study members collected comments, suggestions, and questions from mothers and asked about the questionnaire length and whether the language used was understandable. The questionnaire was revised accordingly and the final version of the questionnaire in Vietnamese was used to collect data.

### Data storing and checking

Each returned questionnaire was checked by a research team member before entering in EpiData version 3.1. Variables were coded to produce a suitable dataset for analysis. A designed form including value ranges and dropdown list were used to minimise missing and incorrect data while entering data.

### Independent and dependent variables

Based on subject-matter knowledge and the availability of data, independent variables were selected including mother’s age, education level, problems during pregnancy and at birth, place of residence, parity, delivery method, type of hospital of birth, infant’s sex and weight at birth, neonatal complications, duration of SSC, received first breastfeeding during the time of SSC.

Dependent variable was the type of feeding the newborn baby received during maternity hospital stay. This variable included two categories: exclusive breastfeeding and non-exclusive breastfeeding.

### Main variables definition

Breastfeeding practices during hospital stay were reported by mothers. Exclusive breastfeeding was determined when an infant received only breast milk, no other liquids or solids are given – not even water, with the exception of oral rehydration solution, or drops/syrups of vitamins, minerals or medicines, according to the WHO definition [25]. In our study, exclusive breastfeeding in hospital was defined as feeding infants only breast milk during maternal hospitalization. First breastfeeding was defined as the initiation of the first breastfeeding during SSC time, before separation from mothers. Neonatal complication was defined when the infant had required resuscitation in the delivery room or needed to transfer to a unit for intensive care. SSC practice was defined as placing the baby SSC with mother right after birth. “Qualified SSC” practice was defined as the satisfaction of both of the following criteria: the duration of SSC of > 90 min and the initiation of the first breastfeeding during SSC time before separation from mother. The definition of “Qualified SSC” was based on the main features of EENC [13] and a recent WHO study found that at least 90 min of uninterrupted SSC maximise the chance of early and exclusive breastfeeding [18]. The terms “urban” and “rural” in this study were used with regard to the place of mother’s residence. Urban was defined as mother’s residence in wards, rural was defined as mother’s residence in commune. This classification based on the General Statistics Office of Vietnam 2019 and the ward was identified as “more developed” than the commune, but not a significant disparity.

### Data analysis

For descriptive analysis, categorical variables were presented with frequency and percentage, while continuous variables were presented with mean and standard deviation (SD) or median, range and interquartile range (IQR) if the distribution was not normal. Multivariable logistic regression with complete-case analysis was used to identify factors associated with exclusive breastfeeding during maternity hospital stay and adjusted odds ratio (aOR) with 95% confidence interval (CI) was expressed. A *p* value of < 0.05 was considered as statistical significance. All analyses were performed using the statistical software R version 3.6.1 (R Foundation for Statistical Computing, Vienna, Austria).

## Results

### Response rate

During the study period, 1812 mothers participated in this study. There were 59 mothers who refused to join the study, corresponding to a response rate of 96.8%. The main reasons for not participating in the study were not having time, the babies crying, babies sleeping, reluctance, and fear of COVID-19.

### Maternal and infant characteristics

Detailed maternal and infant characteristics are presented in Table 1. Nearly 85% of the mothers had education level from high school to postgraduate. There were 49.6% of the infants were delivered by C-section and 64.7% of the infants were delivered in central/provincial hospitals.

**Table 1:** Demographic characteristic, skin to skin contact and breastfeeding practices, Vietnam, 2021

Characteristics	**n**	**Summary statistics (*N* = 1812)**
**Maternal characteristics**		
Age (years), mean ± SD	1801	29.7 ± 5.1
Education qualification	1805	
- Primary school		25 (1.4)
- Secondary school		252 (14.0)
- High school/intermediate degree		982 (54.4)
- University/postgraduate		546 (30.2)
Complication during pregnancy and at birth	1810	170 (9.4)
Place of residence classified as rural	1749	801 (45.8)
Parity, mean ± SD	1809	1.9 (1.2)
**Infant characteristics**		
Sex, male	1806	929 (51.4)
Birth weight (gram), mean ± SD	1810	3212 ± 410
Age (month), mean ± SD	1810	9.7 ± 6.8
C-section	1808	896 (49.6)
Hospital of birth	1799	
- Central/Provincial		1164 (64.7)
- District		480 (26.7)
- Private		147 (8.2)
- Commune		8 (0.4)
Complication at birth	1793	
- Required resuscitation in delivery room		63 (3.5)
- Admission to NICU		46 (2.6)
**Skin-to-skin contact**	1811	
- No skin-to-skin contact		204 (11.3)
- Having skin-to-skin contact^a^		1607 (88.7)
○ < 15 min		524 (32.7)
○ 15–90 min		727 (45.4)
○ > 90 min		351 (21.9)
Qualified skin-to-skin contact		301 (18.8)
**Breastfeeding practices**		
Had a first breastfeed during skin-to-skin contact		894 (55.7)
Exclusive breastfeeding		846 (46.7)
-Breastfeeding		1755 (96.9)
-Formula		742 (40.9)
-Colostrum milk powder		332 (18.3)
-Vitamin/ Medicines		157 (8.7)
-Water		74 (4.1)
-Fruit juices/ Honey		11 (0.6)

*NICU* Neonatal Intensive Care Unit, *SD* Standard Deviation

^a^5 mothers lacking information on SSC time

### Skin-to-skin contact practice

**Fig. 2:**
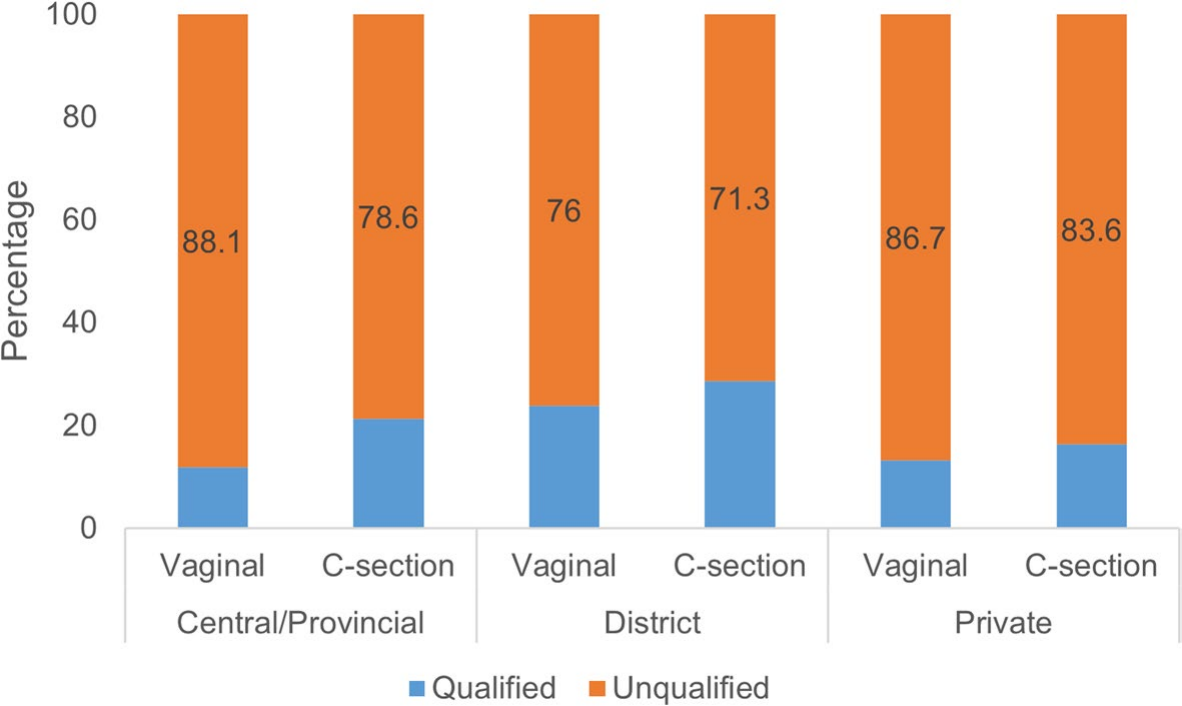
Prevalence of qualified skin-to-skin contact by mode of delivery in different type of hospitals, Vietnam, 2021

Overall, 88.7% of all mothers had SSC with their infants after birth. Prevalence of SSC was significantly higher in district hospitals compared to central/provincial hospitals (p < 0.001). The most common duration of SSC was 15–90 min (45.4%), followed by < 15 min (32.7%) and > 90 min (21.9%). Qualified SSC was identified in 18.8% of all mothers. The C-section group had a higher proportion of SSC than vaginal delivery group in all central/provincial, district and private hospitals (Fig. 2). The highest prevalence of qualified SSC were in district hospital (26.0%).

### Breastfeeding practice

**Fig. 3:**
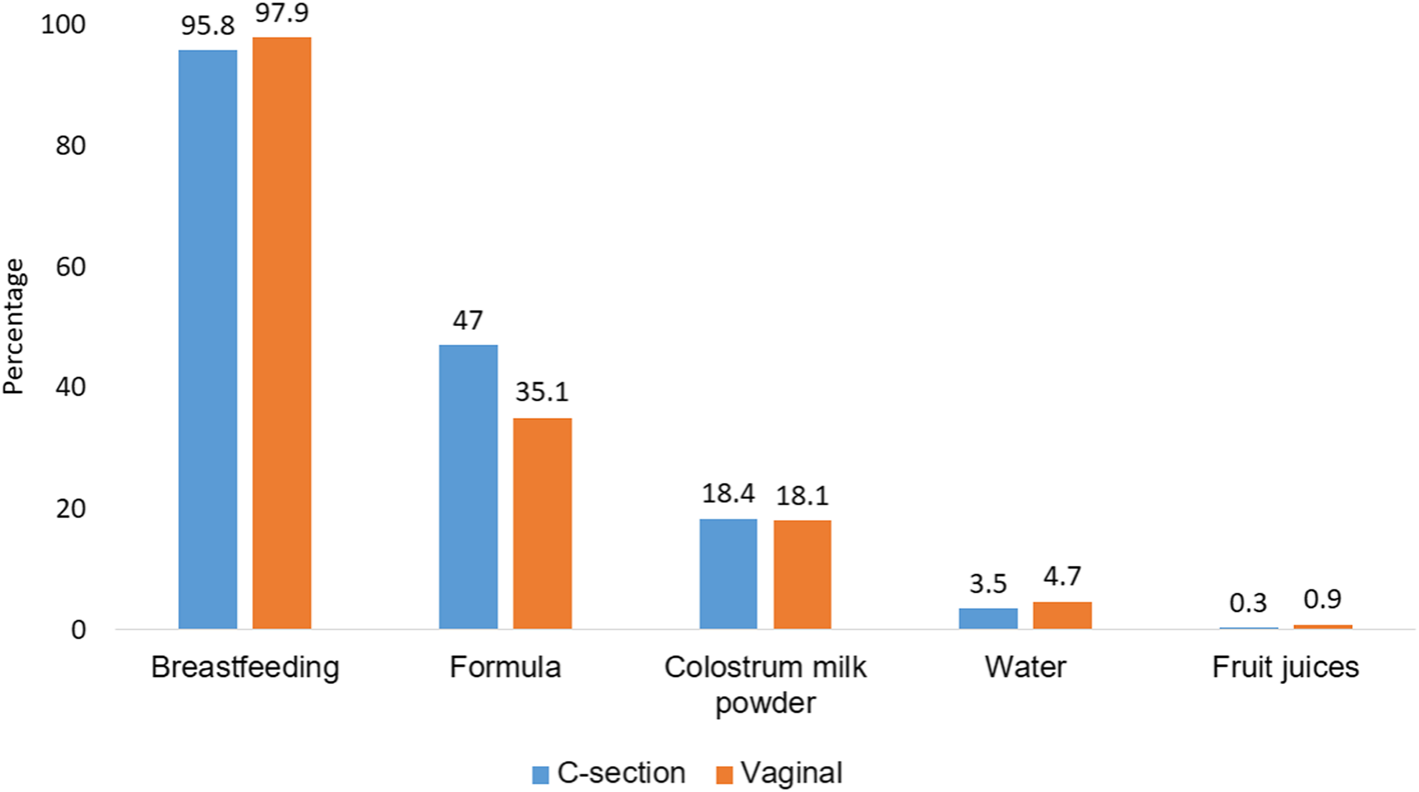
Breastfeeding practice by mode of delivery, Vietnam, 2021

There were 96.9% (*n* = 1755) of mothers breastfed their infants and 40.9% (*n* = 742) provided them with formula during maternal hospital stay. Overall, the exclusive breastfeeding rate was 46.7% (*n* = 846). The exclusive breastfeeding rate was significantly higher in infants born by vaginal delivery than C-section (*p* < 0.0001). The C-section and vaginal delivery groups had similar proportion of breastfeeding (95.8% vs 97.9%, *p* = 0.3) but the C-section group had higher proportion of using formula milk (47% vs 35.1%, *p* < 0.001) (Fig. 3). There were 332 mothers (18.3%) fed their infants with colostrum milk powder which was balanced between the C-section and vaginal delivery groups.

### Duration of skin-to-skin contact associated with increased exclusive breastfeeding rate

In multivariate logistic regression analysis, after adjusting for maternal and infant characteristics in Table 2 (mother’s age and education level, having complication during pregnancy and delivery, place of residence, parity, mode of delivery, hospital of birth, infant’s sex, birth weight and complication at birth), significant association was found between the duration of SSC during maternal hospitalization and the rate of exclusive breastfeeding. The longer early SSC mothers and infants, the more likely that the infants were breastfed exclusively during hospital stay. Compared with infants without SSC, the prevalence of exclusive breastfeeding was higher in infants who experienced SSC for 15–90 min (adjusted odds ratio [aOR], 95% confidence interval [95%-CI]: 2.62 [1.61–4.27]) and more than 90 min (aOR [95%-CI]: 5.98 [3.48–10.28]).

**Table 2:** Factors associated to exclusive breastfeeding at hospital after birth

Characteristics	Non-exclusive (*n* = 966)	Exclusive (*n* = 846)	Unadjusted OR (95% CI)	*P* value	Adjusted OR (95% CI)	*P* value
Age of mother	29.40 ± 4.96	30.04 ± 5.26	1.02 (1.01 to 1.04)	0.009	1.00(0.98 to 1.03)	0.500
Mother education level						
- Primary & Secondary	109 (39.4)	168 (60.6)	Reference		Reference	
- High school/ Intermediate degree	541 (55.1)	441 (44.9)	0.53 (0.4 to 0.69)	< 0.001	0.58 (0.42 to 0.82)	0.002
- University/ Postgraduate	312 (57.1)	234 (42.9)	0.49 (0.36 to 0.65)	< 0.001	0.74 (0.51 to 1.09)	0.125
Complication during pregnancy & at birth						
- No	874 (53.3)	766 (46.7)	Reference		Reference	
- Yes	91 (53.5)	79 (46.5)	0.99 (0.72 to 1.35)	0.953	0.77 (0.52 to 1.14)	0.185
Place of residence						
- Urban	520 (54.9)	428 (45.1)	Reference		Reference	
- Rural	419 (52.3)	382 (47.7)	1.11 (0.92 to 1.34)	0.288	0.78 (0.61 to 0.99)	0.043
Parity	1.87 (0.76)	2.02 (0.8)	1.29 (1.15 to 1.46)	< 0.001	1.13 (0.94 to 1.36)	0.190
Mode of delivery						
- C-section	517 (57.7)	379 (42.3)	Reference		Reference	
- Vaginal delivery	446 (48.9)	466 (51.1)	1.43 (1.18 to 1.72)	< 0.001	1.22 (0.96 to 1.55)	0.099
Hospital of birth						
- Central/Provincial	703 (60.4)	461 (39.6)	Reference		Reference	
- District	170 (35.4)	310 (64.6)	2.78 (2.23 to 3.47)	< 0.001	2.35 (1.79 to 3.09)	< 0.001
- Private	80 (54.4)	67 (45.6)	1.28 (0.90 to 1.8)	0.165	0.99 (0.65 to 1.50)	0.972
Infant’s sex (male)						
-Female	455 (51.9)	422 (48.1)	Reference		Reference	
-Male	506 (54.5)	423 (45.5)	0.9 (0.75 to 1.08)	0.271	0.92 (0.73 to 1.16)	0.488
Infant’s birth weight	3.21 (0.42)	3.21 (0.4)	0.99 (0.79 to 1.24)	0.915	0.79 (0.59 to 1.06)	0.111
Infant’s complication at birth						
-No			Reference		Reference	
-Yes	67 (7.0)	42 (5.0)	0.69 (0.47 to 1.03)	0.069	1.15 (0.69,1.91)	0.593
Skin to skin contact practice (SSC)						
- No SSC	175 (84.7)	29 (15.3)	Reference		Reference	
- < 15 min SSC	377 (71.9)	147 (28.1)	2.16 (1.41 to 3.29)	< 0.001	1.48 (0.92 to 2.4)	0.108
- 15–90 min SSC	333 (45.8)	394 (54.2)	6.54 (4.37 to 9.80)	< 0.001	2.62 (1.61 to 4.27)	< 0.001
- > 90 min SSC	78 (22.2)	273 (77.8)	19.36 (12.31 to 30.45)	< 0.001	5.98 (3.48 to 10.28)	< 0.001
First breastfeeding during SSC	270 (30.3)	622 (69.7)	7.15 (5.81 to 8.80)	< 0.001	4.24 (3.28 to 5.47)	< 0.001

### Other factors associated with exclusive breastfeeding rate

After adjusting for various explanatory variables, initiation of first breastfeeding during SSC time was associated with increased rate of exclusive breastfeeding with aOR of 4.24 (95% confidence interval (CI) 3.28 to 5.47). Another factor increased the odds of exclusive breastfeeding was giving birth in district hospitals. On the other hand, mother education level as high school/intermediate and place of residence classified as rural decreased odds of exclusive breastfeeding (Table 2).

## Discussion

Since 2014, Vietnam was among eight priority countries in the Western Pacific Region that has been supported by WHO to introduce, sustain and scale-up EENC [26]. In 2017, 88% of hospitals in the country have adopted EENC. Our study showed a high prevalence of babies (88.7%) receiving early SSC, but only 18.8% of the babies received qualified SSC. Another key finding of the current research is that the exclusive breastfeeding rate during maternal hospital stay was 46.7%. Multivariable logistic regression identified six factors associated with prevalence of exclusive breastfeeding rate included mother’s education level, place of residence, level of hospital where providing childbirth services, duration of SSC and initiation of first breastfeeding during SSC.

In our study, most infants experienced SSC, consistent with previous reports of the WHO for the Western Pacific Region [26]. This rate, however, showed an impressive improvement of SSC practice to those reported by WHO for Vietnam in 2017 with 59% [26]. Early SSC is a key component of a package of simple evidence-based interventions delivered around the time of birth. It starts in the first minute of life after immediate and thorough drying and assessment of the infants. At least ninety minutes of uninterrupted SSC between mothers and newborns maximizes the chance for infants to be ready to breastfeed [16, 18]. Readiness to breastfeed differs considerably from infant to infant. A longer duration of uninterrupted SSC, the higher possibility of infant completing the first breastfeed [17, 27]. Previous studies have indicated that early initiation of breastfeeding increased the likelihood of exclusive breastfeeding. Our finding regarding the relationship between duration of SSC and prevalence of exclusive breastfeeding during hospital stay is in line with previous results [7, 17, 18]. The optimal SSC duration after birth has not yet been established [18]. In the current study, SSC of 15–90 min was associated with increased prevalence of exclusive breastfeeding compared with non-SSC. However, the most significant improvement in prevalence of exclusive breastfeeding was found when SSC duration was over 90 min.

Despite the fact that uninterrupted SSC for at least 90 min is highly recommended, only about 20% of the infants in our study had experienced > 90 min contact with their mothers. As a result of that, less than one fifth of the infants had received > 90 min of SSC and completed first breastfeeding before separation which was defined as qualified SSC in our study. In 2017, WHO reported a prevalence of prolonged (≥ 90 min) SSC in national hospital was 76% and 25% in subnational hospitals in Vietnam [26]. Our finding, in contrast, showed a lower prevalence of prolonged SSC in central/provincial hospitals than in district hospitals. Over-crowding in central and provincial levels could be a barrier to maintain SSC over 90 min. More importantly, results of both previous reports and current study emphasized the need for improving the quality of SSC including increasing the length of early SSC and completing the first breastfeeding before separation.

Prevalence of exclusive breastfeeding during hospital stay in our study was higher than reported for Vietnam by Le et al. (33%), and Ramoo et al. (7%) [12, 24]. This improvement may be linked to the action at healthcare facilities to limit the use of formula, the strategies for scaling up and improving early SSC practices in a national scale. Our finding also have a similar pattern of breastfeeding behaviour with Le et al. finding that the majority of mothers fed their infants with breast milk during hospital stay. However, formula and colostrum milk powder were still used by nearly half of mothers to feed their infants during the first few days of life. Monitoring and intervention are needed to reduce the use of formula in healthcare facilities.

Factors associated with exclusive breastfeeding during maternity hospital stay is another important finding of our research. Our hypothesis that the duration of uninterrupted SSC had a strong positive dose–response relationship with prevalence of exclusive breastfeeding during hospital stay was confirmed. Moreover, we have included in multivariable logistic regression maternal and infant sociodemographic variables, complication during pregnancy and at birth, mode of delivery, level of hospital at birth, early SSC and initiation of first breastfeeding. After controlling for these factors, level of hospital at birth, place of residence, mother’s education level had impacts on exclusive breastfeeding rate. Our results share a number of similarities with Bramson et al. and Le et al. [12, 17]. SSC between mothers and infants was not uniformly implemented in different levels and types of hospitals [17, 26]. In Vietnam, central and provincial hospitals in large cities are facing a problem of overcrowding with very high occupancy rates. Less overcrowded or not crowded in district hospital may play a part in the effectiveness of SSC including longer duration of SSC. The difference in factors associated with breastfeeding practice between urban and rural was also found in a previous study [12]. The study results provide further evidence to recommend optimal duration of SSC for improving breastfeeding outcomes.

### Strengths and limitations of the study

To our knowledge, this is the first analysis to examine the current practice of SSC in Vietnam and the significant dose–response relationship between increasing duration of SSC and prevalence of exclusive breastfeeding during maternity hospital stay in a lower middle-income country that controlled for potential confounding factors such as maternal and infant’s demographic characteristic, hospital of birth, mode of delivery, problem during pregnancy and at birth of mothers and infants. In addition, the exclusive breastfeeding rate was calculated based on feeding types that mothers reported providing for infants. This is likely to reduce bias resulting from the perception of mothers of the definition of the exclusive breastfeeding.

The study has some limitations. As this study was restricted to mothers who brought their children to the CHC for routine immunisation in two cities of Vietnam, the results may not be generalized for the whole population and do not represent quality of healthcare services. Recall bias is another concern as the study used self-report questionnaire and the data collection were conducted far from birth. Variables such as cultural and the intent to breastfeed, the capability of being able to afford to buy formulas have not been included in our questionnaire.

## Conclusions

This study highlights the suboptimal breastfeeding practice in Vietnam and demonstrate the significant dose–response relationship between increasing duration of SSC and prevalence of exclusive breastfeeding. In addition, the completion of first breastfeeding during SSC time also significantly increased the likelihood of exclusive breastfeeding during hospital stay. To maximise the chance of receiving first breastfeeding and exclusive breastfeeding practice, infants should receive immediate and uninterrupted SSC for 90 min and more. More efforts are needed to accelerate high-quality EENC for improving breastfeeding in Vietnam.

## Data Availability

The datasets generated and/or analysed during the current study are not publicly available, because these data is a subset of a dataset and another manuscripts are drafting based on analysing the dataset, but are available from the corresponding author on reasonable request.

## Abbreviations

EENC: Early Essential Newborn Care
CHC: Commune Health Center
SSC: Skin-to-skin Contact
